# Fecal carriage and molecular epidemiology of carbapenem-resistant Enterobacteriaceae from outpatient children in Shanghai

**DOI:** 10.1186/s12879-019-4298-3

**Published:** 2019-08-01

**Authors:** Fen Pan, Dongxing Tian, Bingjie Wang, Wantong Zhao, Huihong Qin, Tiandong Zhang, Hong Zhang

**Affiliations:** Department of clinical laboratory, Shanghai Children’s Hospital, Shanghai Jiaotong University, Luding Road 355, Putuo District, Shanghai, 200062 China

**Keywords:** Carbapenem-resistant Enterobacteriaceae, Fecal carriage, Molecular epidemiology, Carbapenemase, Outpatient children

## Abstract

**Background:**

Fecal colonization with carbapenem-resistant Enterobacteriaceae (CRE) is a risk factor for bacterial translocation resulting in subsequent endogenous infections. The purpose of this study is to investigate the prevalence of CRE strains colonization in stool samples of outpatient in a tertiary pediatric hospital of Shanghai, China.

**Methods:**

In a retrospective study, fecal samples were consecutively obtained from patients in 2016 and screening test for CRE was conducted by using home-made MacConkey agar. Antimicrobial susceptibility was determined by the broth microdilution method and β-lactamases were characterized by polymerase chain reaction (PCR) assays and DNA sequencing. Multilocus sequence typing (MLST) was performed for the genetic relationships of the isolates.

**Results:**

A total of 880 fecal samples were included for this screening test and 32 CRE strains were identified in 32 non-duplicate fecal samples from 32 children (1.3 ± 1.5 years), with a carriage rate of 3.6%. These strains mainly distributed in *Klebsiella pnuemoniae* (37.5%) and *Escherichia coli* (37.5%). All CRE strains showed high resistance to most of the routinely used antibiotics (> 90%) except for polymyxin B and tigecycline. The *bla*_NDM_ gene was the major carbapenemase gene harbored by gastrointestinal CRE strains, followed by *bla*_KPC-*2*_, *bla*_IMP-26_, and *bla*_IMP-4_. Other β-Lactamase genes including *bla*_CTX-M_, *bla*_SHV_, *bla*_TEM-1_, and *bla*_DHA-1_ were also detected. MLST analysis revealed that various sequence types (STs) were detected in these strains, with ST11 and ST37 being more prevalent in *K.pneumoniae* and ST101 in *E.coli*.

**Conclusions:**

This study revealed the prevalence of CRE fecal carriage in children from outpatient and urgent implementation of infection control measure should be conducted to limit the spread of CRE strains.

## Background

Carbapenems such as imipenem and meropenem are often recommended as antimicrobial agents of the last resort, especially in cases where extended-spectrum β-Lactamase (ESBL) producing organisms involved [1]. Since the KPC1 carbapenem- resistant *Klebsiella pneumoniae* was first isolated in the United States of America [2], the prevalence of carbapenem-resistant Enterobacteriaceae (CRE) has emerged as a public health problem and given rise to epidemic outbreaks in hospital surroundings all around the world [3–5]. According to the CHINET surveillance of bacterial resistance data in China, CRE strains showed high levels of resistance to most antibiotics and the rates of carbapenem resistance in *E. coli* and *K. pneumoniae* had increased from 0 and 2.9% in 2005 to 1 and 13.4% in 2014, respectively [6]. In general, the mechanism of resistance to carbapenems is mainly associated with the production of carbapenemases.

Enterobacteriaceae are a family of bacteria that mainly colonize in normal human intestines. Gastrointestinal tract colonization with CRE strains can be a risk factor for bacterial translocation resulting in subsequent endogenous infections in immunocompromised children [7]. As Das et al. reported that neonates with Gram negative bacilli in the gut had a higher incidence of clinical sepsis than those without these bacteria [8]. The acquisition of mobile genetic elements may be involved in the horizontal transmission. Several studies have been conducted to investigate the fecal carriage characteristics of CRE strains among inpatients [9–11], however, there are few data regarding the prevalence of CRE colonization in community settings, especially in China. Therefore, this research retrospectively aimed to conduct a surveillance of molecular epidemiology of CRE strains colonized in stool samples of outpatient children in a tertiary pediatric hospital of Shanghai, China.

## Methods

### Specimen collection and bacterial screening

This study was retrospectively conducted in 2016 from January to December at Shanghai Children’s Hospital, which is one of largest pediatric hospitals with 700 beds. Epidemiological information including patient demographics, prior hospitalization, previous receipt of antibiotic therapy, and invasive operation during hospitalization was obtained from the medical records of each patient.

Fecal samples were consecutively obtained from patients of ≤18 year old attending in outpatient clinics, who received the fecal culture testing. Approximately 0.5 mg of stool or a rectal swab from each patient was stored in Cary-Blair transport medium (Hopebio, Qingdao, China) in 4 °C condition up to 1 days prior to testing. Screening test of CRE was performed as follows: fecal samples were inoculated in home-made MacConkey agar supplemented with meropenem at 1 μg/ml and then incubated at 35 °C in a 5% CO2 incubator. After incubation for 24-48 h, the growth of at least one colony per agar plate was considered as positive screening result and the strain was further investigated. All CRE strains were stored at − 80 °C in 40% glycerol broth medium for further analysis.

### Identification and antimicrobial susceptibility testing

All CRE bacteria were identified by matrix-assisted laser desorption ionization time of flight (MALDI-TOF) mass spectrometry using MALDI Biotyper (Bruker Daltonik GmbH, Bremen, Germany). Antimicrobial susceptibility testing of all CRE strains against amikacin, cefotaxime, ceftazidime, cefepime, piperacillin-tazobactam, ertapenem, imipenem, meropenem, ciprofloxacin, fosfomycin, aztreonam, colistin, and tigecycline was carried out by using broth microdilution method for determining the minimal inhibitory concentrations (MICs). The breakpoints used for interpretation were recommended by the Clinical and Laboratory Standards Institute (CLSI) 2017 [12]. Quality control was managed by using *E.coli* ATCC 25922. The EUCAST breakpoints were used for colistin. The interpretive criterion for tigecycline was based on the breakpoints of the Food and Drug Administration (FDA).

### Screening for carbapenemase and other resistance genes

Polymerase chain reaction (PCR) was used to detected carbapenemase genes (*bla*_NDM_, *bla*_KPC_, *bla*_IMP_, *bla*_VIM_, *bla*_OXA-48_, *bla*_GES_, *bla*_GIM_, and *bla*_SIM_), ESBL genes (*bla*_CTX-M_, *bla*_TEM_, *bla*_SHV_), AmpC genes (*bla*_DHA_, *bla*_CMY_, *bla*_MOX_, *bla*_EBC_, and *bla*_AAC_), and the colistin resistance gene (*mcr-1*) as previously described [13–15]. Amplicons were sequenced and nucleotide sequences were further analyzed and compared to sequences available at the National Center for Biotechnology Information (NCBI) website (https://blast.ncbi.nlm.nih.gov/Blast.cgi).

### Multilocus sequence typing (MLST)

MLST was performed for the genetic relationship according to the previous protocol described. Seven housekeeping genes including *ropB, gapA, mdh, pgi, phoE, infB,* and *tonB* were amplified and sequenced for *K. pneumoniae* [16]. Alleles and sequence types (STs) were assigned at the Pasteur Institute MLST website (http://www.pasteur.fr/recherche/genopole/PF8/mlst/Kpneumoniae.html). For *E. coli,* the housekeeping genes of *adk, fumC, gyrB, icd, mdh, purA,* and *recA* were amplified and sequenced [17], and STs were then further assigned by using the MLST database (http://mlst.warwick.ac.uk/mlst/dbs/Ecoli). The seven housekeeping genes of *Enterobacter cloacae* (*dnaA, fusA, gyrB, leuS, pyrG, rplB,* and *rpoB*) were also amplified and sequenced [18], and STs were assigned at the MLST website (https://pubmlst.org/ecloacae/).

### Statistical analysis

Statistical analysis was performed by using SPSS 19.0 for Windows (version 19.0; SPSS Inc., Chicago, IL, USA). The quantitative data were expressed as means ± standard deviations (SD). Categorical variables, expressed as numbers and percentages, were compared by the Chi-square or Fisher’s exact test. A value of *P* ≤ 0.05 was considered statistically significant. Antimicrobial resistance data was analyzed by using WHONET 5.6.

## Results

### Screening result and patient information

During the study period, a total of 880 fecal samples from 880 children (1.1 ± 2.2 years) in outpatient clinics were collected for this screening test. 32 non-duplicate samples were detected as being CRE positive with a carriage rate of 3.6% (32/880). The 32 Enterobacteriaceae strains included *K.pneumoniae* (*n* = 12, 37.5%), *E.coli* (*n* = 12, 37.5%), *E.cloacae* (*n* = 6, 18.8%), *Enterobacter aerogenes* (*n* = 1, 3.1%), and *Raoultella planticola* (*n* = 1, 3.1%).

The CRE strains were isolated from 32 individual children (19 male and 13 female) whose mean age was 1.3 ± 1.5 years (rang: 1 m-5y). Among these children, 28.1% (9/32) had a history of prior hospitalization in recent 3 months and these patients also had received therapy with antibiotics (e.g. imipenem, cefperazone-sulbactam, ampicillin/sulbactam, or cefotaxime). Of the 32 CRE carriers and 848 non-CRE carriers identified, 9 (28.1%) and 38 (4.5%) children, respectively, had been hospitalized and received antibiotics within the past 3 months (*P* < 0.05). Among nine previously hospitalized CRE carrier children, only two children had received invasive operation during their hospitalization.

### Antimicrobial susceptibility

The results of the antimicrobial susceptibility testing of the 32 CRE strains are shown in Table 1. All CRE isolates showed high resistance to cephalosporins and carbapenems (> 95%). The rates of susceptibility to amikacin, ciprofloxacin, aztreonam and fosfomycin were 75, 25, 21.6, and 15.6%, respectively. Tigecycline retained excellent activity against all 32 CRE isolates, with a susceptibility rate of 100%, regardless of the species. Additionally, the CRE strains showed a high susceptibility rate to colistin (90.6%). However, the MIC ≥4 μg/mL of colistin was observed in *E.coli* and *E.cloacae* isolates*.*

**Table 1:** Antimicrobial susceptibility profiles of carbapenem-resistant Enterobacteriaceae strains from fecal samples, % (n)

Antibioics	Resistant	Intermediate	Susceptible
Cefotaxime	100 (32)	0 (0)	0 (0)
Ceftazidime	100 (32)	0 (0)	0 (0)
Cefepime	100 (32)	0 (0)	0 (0)
Piperacillin–tazobactam	78.1 (25)	6.3 (2)	15.6 (5)
Ertapenem	96.9 (31)	3.1 (1)	0 (0)
Imipenem	96.9 (31)	3.1 (1)	0 (0)
Meropenem	96.9 (31)	3.1 (1)	0 (0)
Amikacin	25 (8)	0 (0)	75 (24)
Ciprofloxacin	71.9 (23)	3.1 (1)	25 (8)
Fosfomycin	81.2 (26)	3.1 (1)	15.6 (5)
Aztreonam	78.1 (25)	0 (0)	21.9 (7)
Colistin	9.4 (3)	0 (0)	90.6 (29)
Tigecycline	0 (0)	0 (0)	100 (32)

### Carbapenemase and other resistance genes

Among the isolated CRE strains, the NDM-1 type carbapenemase gene (*bla*_NDM-1_) was the most common and detected in 14 strains (43.8%, 14/32), followed by *bla*_NDM-5_ gene (18.8%, 6/32). Other carbapenemase genes including *bla*_KPC-2_, *bla*_IMP-26_, and *bla*_IMP-4_ were also found. The characteristics of the *E.coli* and *K.pneumoniae* isolates are shown in Tables 2 and 3, respectively. For *E.coli* strains, only the *bla*_NDM-1_ and *bla*_NDM-5_ genes were detected, whereas *bla*_NDM-1_, *bla*_NDM-5_, *bla*_KPC-2_, and *bla*_IMP-4_ were found in the *K.pneumoniae* strains. The *bla*_IMP-26_ carbapenemase gene was only observed in *E.cloacae*.

**Table 2:** Characteristics of carbapenem-resistant *Klebsiella pneumoniae* colonized in fecal samples

NO.	Carbapenemase	Other resistance genes	MLST	MIC (μg/mL)												
				AK	FEP	CTX	CAZ	TZP	ETP	MEM	IPM	CIP	ATM	FOS	COL	TGC
16439	NDM-1	CTX-M-14, SHV-2, DHA-1	ST14	≤16	≥256	≥256	≥256	≥256/4	32	128	32	16	128	≥512	1	≤0.125
60006	NDM-1	CTX-M-14	ST11	≥256	128	≥256	≥256	≥256/4	64	128	64	32	≤4	≥256	1	≤0.125
E17	NDM-1	CTX-M-14	ST11	≥256	128	≥256	≥256	256/4	256	256	64	128	≥256	256	1	≤0.125
52084	NDM-1	CTX-M-14, SHV-2, TEM-214	ST37	≥256	128	≥256	≥256	256/4	256	256	64	128	≥256	256	1	≤0.125
53706	IMP-4	CTX-M-14, TEM-1, SHV-11	ST17	≤16	64	128	≥256	32/4	4	16	4	≤1	≤4	≥256	1	0.25
E1	NDM-5	CTX-M-15, SHV-11	ST48	≤16	≥256	≥256	≥256	≥256/4	≥256	≥256	256	≤1	≥256	≥256	1	≤0.125
E3	NDM-5	CTX-M-15, SHV-11	ST792	≤16	≥256	≥256	≥256	≥256/4	128	256	128	4	32	256	1	0.25
E6	NDM-5	CTX-M-15, SHV-11	ST48	≤16	≥256	≥256	≥256	≥256/4	≥256	≥256	≥256	8	128	≥256	1	≤0.125
E8	KPC-2	–	ST11	≥256	≥256	≥256	≥256	≥256/4	128	256	128	64	≥256	≥256	1	0.25
E9	NDM-1	CTX-M-15, SHV-1	ST37	≤16	64	≥256	≥256	≥256/4	32	128	16	≤1	128	≤64	1	≤0.125
E11	KPC-2	–	ST11	≥256	64	128	≥256	≥256/4	128	256	32	64	≥256	≥256	1	≤0.125
E27	KPC-2	CTX-M-65, SHV-1	ST215	≤16	32	128	256	≤16/4	1	4	4	≤1	≤4	256	1	0.25

*AK* amikacin, *CTX* cefotaxime, *CAZ* ceftazidime, *FEP* cefepime, *TZP* piperacillin-tazobactam, *ETP* ertapenem, *IPM* imipenem, *MEM* meropenem, *CIP* ciprofloxacin, *FOS* fosfomycin, *ATM* aztreonam, *COL* colistin, *TGC* tigecycline

**Table 3:** Characteristics of carbapenem-resistant *Escherichia coli* colonized in fecal samples

NO.	Carbapenemase	Other resistance genes	MLST	MIC (μg/mL)												
				AK	FEP	CTX	CAZ	TZP	ETP	MEM	IPM	CIP	ATM	FOS	COL	TGC
16366	–	CTX-M-14	ST7151	≤16	≥256	≥256	128	≤16/4	16	8	16	16	128	≥512	1	≤0.125
16495	NDM-1	CTX-M-14	ST101	≤16	≥256	≥256	64	≤16/4	16	8	16	16	128	≥512	1	≤0.125
24354	NDM-5	CTX-M-14	ST167	≤16	32	≥256	64	≤16/4	32	16	32	16	128	≥256	1	≤0.125
27291	–	CTX-M-14, DHA-1	ST2003	≤16	≥256	≥256	256	256/4	32	8	16	16	128	≥256	1	≤0.125
60286	NDM-1	CTX-M-14	ST10	≤16	64	≥256	≥256	≥256/4	32	128	32	32	64	≥256	1	≤0.125
60066	NDM-5	CTX-M-15	ST457	≥256	128	≥256	≥256	≥256/4	64	128	64	32	≤4	≥256	1	≤0.125
60883	NDM-1	CTX-M-14, DHA-1	ST101	≤16	64	≥256	≥256	≥256/4	256	128	32	16	≥256	≥256	1	≤0.125
44376	NDM-1	CTX-M-14	ST648	≤16	≥256	≥256	≥256	≥256/4	256	256	64	64	≤4	≤64	1	≤0.125
48556	NDM-1	CTX-M-14, DHA-1	ST101	≤16	128	≥256	≥256	≥256/4	8	16	8	8	128	≤64	4	0.25
50831	NDM-1	CTX-M-14	ST101	≥256	≥256	≥256	≥256	≥256/4	64	64	32	128	≥256	≥256	≥4	≤0.125
55414	–	CTX-M-14	ST12	≤16	≥256	≥256	≥256	≥256/4	16	64	32	64	32	≤64	1	≤0.125
E10	NDM-5	CTX-M-15, TEM-1	ST68	≤16	64	≥256	≥256	≥256/4	128	128	64	2	≤4	≤64	1	≤0.125

*AK* amikacin, *CTX* cefotaxime, *CAZ* ceftazidime, *FEP* cefepime, *TZP* piperacillin-tazobactam, *ETP* ertapenem, *IPM* imipenem, *MEM* meropenem, *CIP* ciprofloxacin, *FOS* fosfomycin, *ATM* aztreonam, *COL* colistin, *TGC* tigecycline

A majority of CRE strains simultaneously harbored ESBL genes. The dominant ESBL gene was *bla*_CTX-M_. The common subtypes of *bla*_CTX-M_ in these strains were *bla*_CTX-M-14_ (*n* = 19), *bla*_CTX-M-15_ (*n* = 9), and *bla*_CTX-M-65_ (*n* = 1). Other ESBL genes including *bla*_SHV-1_, *bla*_SHV-2_, *bla*_SHV-11_, *bla*_TEM-1_, and *bla*_TEM-214_ were also detected. The *bla*_DHA-1_ gene, which is an AmpC gene, was found in five isolates. No *mcr-1* gene was identified in all strains.

### Characteristics of STs

MLST analysis revealed that STs distributed dispersedly in different species colonized in fecal samples from community and a total of twenty distinct STs were identified among 32 CRE isolates, with various STs in different species. As depicted in Table 3, nine STs were observed among carbapenem-resistant *E.coli* and ST101 was main type, accounting for 33.3%. We also found seven STs in carbapenem-resistant *K.pneumoniae* isolates, with ST11 being the major type (Table 2). Then four STs were identified in the six *E.cloacae* isolates.

## Discussion

Investigation of the fecal carriage prevalence of CRE among outpatients from the community setting can help us to better understand the origin of CRE isolates responsible for outbreak events and contribute to control CRE dissemination. Our study displayed that the rate of colonization with CRE in fecal sample collected from children in outpatient visits was 3.6%, which was lower than what had been reported in patients from community setting in other countries [10, 19]. Several reasons contribute to this phenomenon of the colonization of CRE isolates in rectal gastrointestinal tract. Firstly, further analysis of patient information of CRE carriers showed that 28.1% of them had a history of prior hospitalization in recent 3 months and also had received therapy with antibiotics, but only 4.5% of non-carriers had. Previous studies reported that exposure to hospital setting or/and antimicrobial agents might increase the risk of colonization and it would be easier to acquire CRE isolates, which may also explain the occurrence in community-onset cases [20, 21]. However, despite the previous studies showed an increased risk of CRE carriage with hospital exposure, there is no significance between the min, max and mean times of hospitalization for the CRE and non-CRE groups with recent hospital exposure in this study. What is more, many outpatients receive antimicrobial treatment without the advice or prescription of a physician or other trained health care provider. Last but not least, CRE strains could spread via physical contact with other people and have the propensity to acquire genetic materials mostly in the form of plasmids and transposons through horizontal gene transfer [22]. Colonization with CRE strain plays an important role in transmission of the orgnism through horizontal gene transfer in health care settings [22].

The surveillance definition for CRE are designed that Enterobacteriaceae are resistant to imipenem, meropenem, doripenem, or ertapenem or is document that the isolate possess a carbapenemase and CRE are reported to be commonly identified in *K.pneumoniae*, *E.coli*, and *E.cloacae* [23, 24]. The major resistance mechanism of CRE is the production of carbapenemases such as NDM, KPC, and IMP, which were also observed in this study. The NDM type, including NDM-1 and NDM-5, is found to be the key carbapenemase responsible for mediating development of the carbapenem resistance phenotypes in children [3, 25]. NDM-1 and its minor variants, a class B carbapenemase first clinically isolated from a patient at a hospital in New Delhi, India, has since been identified all over the world and only detected in *E.coli* and *K.pneumoniae* [26–29]. Consistent with the above findings, most of the *E.coli* and *K.pneumoniae* colonized CREs in the fecal samples in the present study harbored the *bla*_NDM_ gene and a previous study in our hospital also has reported the outbreak of CRE strains caused by NDM-1 producing *K.pneumoniae* among neonates [3]. NDM-1 producing *K.pneumoniae* are highly resistant pathogens with no effective beta-lactams, including recent ones such as ceftolozane tazobactam and ceftazidime avibactam and the only one that works is aztreonam [30]. Additionally, only three *K.pneumoniae* strains carried *bla*_KPC-2_ gene, which was mostly prevalent in adults population [31], were found in this study. IMP type carbapenemases (IMP-4 and IMP-26) were observed in *E.cloacae* and *K.pneumoniae*, but not in *E.coli.* However, it is remarkable that no carbapenemase resistance genes were detected in 9.4% (3/32) of the CRE strains. This finding suggests that a new mechanism might contribute to the resistance to carbapenems and further studies into this mechanism should be conducted.

Molecular analysis revealed a high level of genetic diversity, including ST101, ST11, ST37, and ST48, and several STs are clearly related to specific bacteria, and are prevalent all over the world. The most common STs of CRE in *K.pneumoniae* were ST11 and ST37*.* Interestingly, the carbapenem-resistant *K.pneumoniae* harboring *bla*_KPC-2_ belonged to ST11, which exhibited signs of multi-clonal dissemination and caused a series of outbreaks in China [32, 33]. Additionally, another carbapenem- resistant *K.pneumoniae* harboring *bla*_NDM-1_ belonged to ST37 and this ST had been identified previously during an outbreak of this phenotype in our hospital [3]. Clonally diverse carbapenem-resistant *E.coli* (ST405, ST131, ST156 and ST101) have been reported globally, and ST101 was the predominant ST isolated in this study. The ST101 type of *E.coli* produced NDM-1 carbapenemase, a phenotype that has been linked to nosocomial transmission in Korea [34].

Importantly, children with CRE strains in fecal samples are considered as a high risk group by World Health Organization (WHO), which can spread CRE by intimate contact and travel [35]. The origin of CRE isolates in these children remains unknown and it is the main limitation that we don’t know if the source of the described CRE carriage in these children is the result of transmission in the community or in the hospital. Screening for CRE at discharge may provide an explanation for this hypothesis. Exposure to antimicrobial agents in both the hospital setting and environments is always the key to acquisition. Also we know that children are at high risk for multi-drug organism carriage and further studies should be carried out to evaluate the phenomenon of the original source of CRE strains.

## Conclusions

In summary, the current data reveal the prevalence of CRE colonization in fecal sample from pediatric outpatients and strategies to control the dissemination of antimicrobial resistant isolates from stool to other sterilized sites should be developed. Children who had hospitalization exposure should be screened for CRE at discharge, which may help to definitively establish where acquisition of CRE is truly occurring and decrease the occurrence and transmission of CRE in children group.

## Data Availability

Please contact corresponding author for data requests.

## Abbreviations

CLSI: Clinical laboratory standard institute
CRE: Carbapenem-resistant Enterobacteriaceae
ESBL: Extended-spectrum β-Lactamase
MALDI-TOF: Matrix-assisted laser desorption ionization time of flight
MLST: Multilocus sequence typing
PCR: Polymerase chain reaction
ST: Sequence type
